# Transmission of *Anaplasma phagocytophilum* (Foggie, 1949) by *Ixodes ricinus* (Linnaeus, 1758) ticks feeding on dogs and artificial membranes

**DOI:** 10.1186/s13071-019-3396-9

**Published:** 2019-03-26

**Authors:** Josephus J. Fourie, Alec Evans, Michel Labuschagne, Dionne Crafford, Maxime Madder, Matthias Pollmeier, Bettina Schunack

**Affiliations:** 1 Clinvet International (Pty) Ltd, Bloemfontein, South Africa; 2 Clinvet SA Morocco, Mohammedia, Morocco; 3 Clinglobal, Black River, Mauritius; 4 Bayer Animal Health GmbH, Monheim, Germany

**Keywords:** *Anaplasma phagocytophilum*, *Ixodes ricinus*, Transmission, *In vivo*, *In vitro*, Dogs

## Abstract

**Background:**

The interplay of speed of activity of acaricidal products and tick-borne pathogen transmission time is the major driver for disease prevention. This study aimed to investigate the time required for transmission of *Anaplasma phagocytophilum* by adult *Ixodes ricinus* ticks *in vivo* on dogs, and to confirm the time required for transmission observed *in vivo*, *in vitro*.

**Methods:**

Nymphs of *I. ricinus* were experimentally infected with an *A. phagocytophilum* strain of canine origin. Dogs were allocated to 6 groups of 3 dogs each. Groups 1–5 were infested with 50 *A. phagocytophilum*-infected female adult ticks on Day 0. Ticks were removed post-infestation at 3, 6, 12, 24 and 48 h. Dogs in Group 6 were infested with 60 *A. phagocytophilum*-infected female adult ticks (left on dogs until engorged). Dogs were observed daily for general health and clinically examined on Day 0, and weekly from Day 14. Blood was collected for qPCR and serological analysis on Day 0 (pre-challenge) and weekly thereafter. In the *in vitro* study each artificial feeding chamber was seeded with 10 adult ticks (5 male/5 female), attachment assessed, and blood pools sampled for qPCR at 6 h intervals up to 72 h after first tick attachment.

**Results:**

*Anaplasma phagocytophilum* specific antibodies and DNA were detected in all 3 dogs in Group 6. No *A. phagocytophilum*-specific antibodies or DNA were detected in any dogs in Groups 1–5. All dogs remained healthy. Female tick attachment in 60 artificial feeding chambers over 72 h ranged between 20–60%. *Anaplasma phagocytophilum* DNA was detected in the blood collected from 5% of chambers sampled at 6 h, with the highest number of positive samples (16.3%) observed at 36 h.

**Conclusions:**

Transmission of *A. phagocytophilum* by *I. ricinus* ticks starts within a few hours after attachment but establishment of infections in dogs is apparently dependent on a minimum inoculation dose that was only observed when ticks attached for greater than 48 h. These findings highlight the need for acaricidal products to exert a repellent and/or rapid killing effect on ticks to forestall transmission and subsequent disease.

## Background

*Anaplasma phagocytophilum*, transmitted by ixodid ticks, is regarded as an emerging pathogen of humans, horses and dogs worldwide. In dogs, this pathogen is the causative agent of canine granulocytic anaplasmosis, a disease with nonspecific clinical signs like lethargy and reduced activity, fever and inappetence most frequently observed [1–5]. Studies suggest that multiple strains of *A. phagocytophilum* may be circulating in wild and domestic animal populations, with differential host tropisms and pathogenicity and that co-infections with other tick-borne pathogens may occur, especially *Borrelia burgdorferi* [6]. Due to the health risks posed to dogs by tick-borne diseases, the importance of acaricidal products to protect against tick infestations and the pathogens they transmit is a growing concern worldwide. As such, various studies have been conducted evaluating the ability of acaricidal products to forestall transmission of tick-borne pathogens like *Babesia* spp., *Ehrlichia canis*, *B. burgdorferi* and *A. phagocytophilum* [7–12].

The protective ability of an acaricidal product to prevent transmission of a tick-borne pathogen can be explained by several properties of the acaricidal molecule: a repellent/irritant effect inhibiting tick infestation and attachment, a neuro-hormonal disruption of tick attachment and intake of the blood meal, and/or a quick speed of kill before transmission can occur [13]. Moreover, the relevance of these properties in preventing transmission is dependent on the speed of transmission of the specific pathogen by its tick vector. These transmission times are highly variable and can be slow as is the case for *Babesia* spp. because of the 36–48 h minimum duration of attachment and initial feeding required for sporogony, or fast as is the case for tick-transmitted bacteria like *E. canis* (within 3 h) or viruses like Powassan virus (within 15 min) [14–16]. Moreover, transmission times can also be shortened once a tick has taken a blood meal and feeding is interrupted, as demonstrated for male *Dermacentor reticulatus* infected with *B. canis*, shortening the attachment time needed for transmission from a minimum of 36–48 h to less than 8 h [17].

Knowledge of the speed at which a specific pathogen is transmitted by its tick vector is therefore imperative to determine the “grace” period within which a specific acaricidal product could be able to prevent transmission. Although the speed of transmission has been investigated for various tick-transmitted pathogens like *B. canis* and *E. canis* in dogs, very little information is available on the speed of transmission of *A. phagocytophilum* by *Ixodes ricinus* ticks [16, 17]. Various authors have cited that ticks must attach for 36–48 h for transmission of *A. phagocytophilum* to occur based on research done by Hodzic et al. [18] and Katavolos et al. [19]. Although both these studies provided valuable insights into the transmission dynamics of *A. phagocytophilum*, both were conducted using nymphal *Ixodes scapularis* ticks on mice and with a human *Ehrlichia phagocytophila* isolate, later reclassified as *A. phagocytophilum* [20]. Considering that *A. phagocytophilum* is regarded as an emerging pathogen of dogs worldwide, it is important to understand the transmission dynamics of this pathogen in dogs in more detail [6].

The aim of this study was to determine the time required for transmission of *A. phagocytophilum* by adult *I. ricinus* ticks *in vivo* on dogs, and to confirm the time required for transmission observed *in vivo* in an *in vitro* experiment using artificial feeding membranes.

## Methods

### *Anaplasma phagocytophilum* strain

The *A. phagocytophilum* strain used (“TIBA strain”) was isolated in June 2015 from a clinical case (dog) in Terschelling, the Netherlands. Amplification of the *ank* gene was performed as described by Massung et al. [21], followed by Sanger sequencing of the PCR product on both strands. The assembled sequence was subjected to BLAST analysis and 142 sequences from GenBank (having a > 99% coverage of the query sequence) were used in a multiple alignment using MAFFT, followed by Bayesian inference analysis (HKY85 substitution model; 2 heated chains with a chain length of 4,000,000; sampling frequency of 1000; 25% ‛burn-inʼ) using GU236882 as the outgroup.

### Infection of *Ixodes ricinus* ticks with *Anaplasma phagocytophilum*

*Ixodes ricinus* nymphs were fed on a sheep infected with the “TIBA strain” of *A. phagocytophilum* described above. Sheep were confirmed infected using qPCR analysis of blood. Nymphs were left to feed until engorged, after which the detached fully engorged nymphs were collected and allowed to moult at 20 °C, 90% relative humidity (RH) and 16 h:8 h Light:Dark photoperiod.

The methodology described above was used to breed 3 infected tick batches; 2 tick batches were used for the *in vivo* study and 1 tick batch for the *in vitro* study. Successful infection of adult *I. ricinus* ticks was confirmed by qPCR on a sample of 50 ticks (25 male/25 female) taken from the each tick batch collected from the donor sheep.

### Dog study design

The *in vivo* component of the study was conducted at Clinvet Morocco with 6 groups of 3 dogs each. At the time of enrolment all dogs were between 2–6 years of age, and weighed between 12–21 kg. All dogs were healthy based on clinical examination by a veterinarian and seronegative for *A. phagocytophilum* antibodies prior to inclusion in the study. The study dogs had not been treated with any acaricidal product for 12 weeks prior to the first tick challenge. Dogs were individually housed in indoor cages fitted with a sleeping bench, were fed a commercial dog food once daily and provided water *ad libitum*.

### Tick infestation, attachment observations, counts and removal

To allow for an accurate assessment of tick attachment and removal, ticks were infested in chambers fitted to the skin of the dogs in Groups 1–5. In these groups, each dog was fitted with 2 feeding chambers (10 cm in diameter) on the lateral shoulder. The chambers were joined to the shaved skin of dogs using cyanoacrylate adhesive applied to the chambers immediately before placement. Pressure was applied for at least 30 s after joining the feeding chambers to the skin. Dogs were fitted with Elizabethan collars from the time of chamber attachment to the time of removal to minimize the risk of damaging or dislodging the chambers containing the ticks. At each assessment period, the chambers fitted to each dog and the site of fitment were examined for any abnormality. All chambers were removed from the dogs after completion of assessments using DMSO to dissolve the cyanoacrylate adhesive.

Each dog in Groups 1–5 was infested with 50 female ticks (25 ticks per chamber) with a confirmed infectivity of 37%, whilst dogs in Group 6 received a full body infestation with 60 female *I. ricinus* ticks with a confirmed infectivity of 21%.

At 3 h after tick infestation, all non-attached ticks were removed from each feeding chamber and counted. At 3, 6, 12, 24 and 48 h, all remaining ticks were removed, sexed, counted, categorized based on attachments status and viability for Groups 1–5, respectively. All attached female ticks were assessed by qPCR for *A. phagocytophilum* DNA to confirm infectivity. Male ticks were discarded. Ticks infested on dogs in Group 6 were allowed to feed until engorged and all engorged detached ticks were collected from the cage environment.

### Monitoring of dogs for general health and *Anaplasma phagocytophilum* infection

All dogs were observed daily for general health and clinically examined by a veterinarian on Day 0, and weekly from Day 14 up to study completion. Clinical examinations included general appearance by body system, respiration rate, heart rate and body temperature. Particular attention was given to the most common clinical manifestations of anaplasmosis, which included lethargy and reduced activity, fever and inappetence. Rectal body temperatures were recorded daily from Day 5 to study completion (Day 63 for Groups 1–5 and Day 42 for Group 6). Group 6 terminated on Day 42 as all dogs had already presented with 2 positive serology results by this day. At least 3.5 ml of blood was collected in EDTA tubes for qPCR and serological analysis on Day 0 (prior to tick challenge) and weekly thereafter until study completion.

### Laboratory assays

Blood collected from dogs (200 µl) was directly subjected to genomic DNA isolation using the NucleoMag Vet kit (Macherey-Nagel, Dűren, Germany) using a KingFisher Flex 96 instrument (Thermo Fisher Scientific, Waltham, USA). The DNA isolation procedure was modified to include a post-lysis RNase A treatment (10 µl of 20 mg/ml RNase A per sample) for 30 min at room temperature. DNA was recovered using 100 µl of elution buffer and quantified spectrophotometrically and assessed using agarose gel electrophoresis. A total of 2 µl of DNA served as template for subsequence qPCR detection. *Anaplasma phagocytophilum*-specific qPCR primers and probe targeting the MSP2 region were used to detect the presence of *A. phagocytophilum* DNA in the extract [22]. *Anaplasma phagocytophilum* MSP2 quantification was performed for dogs in Group 6 (*A. phagocytophilum* infected female adult ticks were left on the dogs until engorged). SsoAdvanced^TM^ Universal Probes Supermix (Bio-Rad, Hercules, USA) was used in a 20 µl reaction volume containing 300 nM each primer and 200 nM probe, followed by thermal cycling at 95 °C for 10 min and 40 cycles of 95 °C for 15 s and 60 °C for 1 min. Control reactions included positive, negative, extraction and no template controls, as well as an internal amplification control to limit false negative results.

Tick infectivity was determined by homogenizing individual ticks using high density zirconium oxide beads, followed by genomic DNA isolation and qPCR detection as described above.

For serology, 3 drops of whole blood were transferred to a micro tube for the detection of antibodies to *A. phagocytophilum* using a SNAP^®^ 4Dx^®^ Plus test (IDEXX Laboratories Inc., Westbrook, ME, USA). The samples were processed according to the manufacturer’s instructions.

### *In vitro* study

**Fig. 1:**
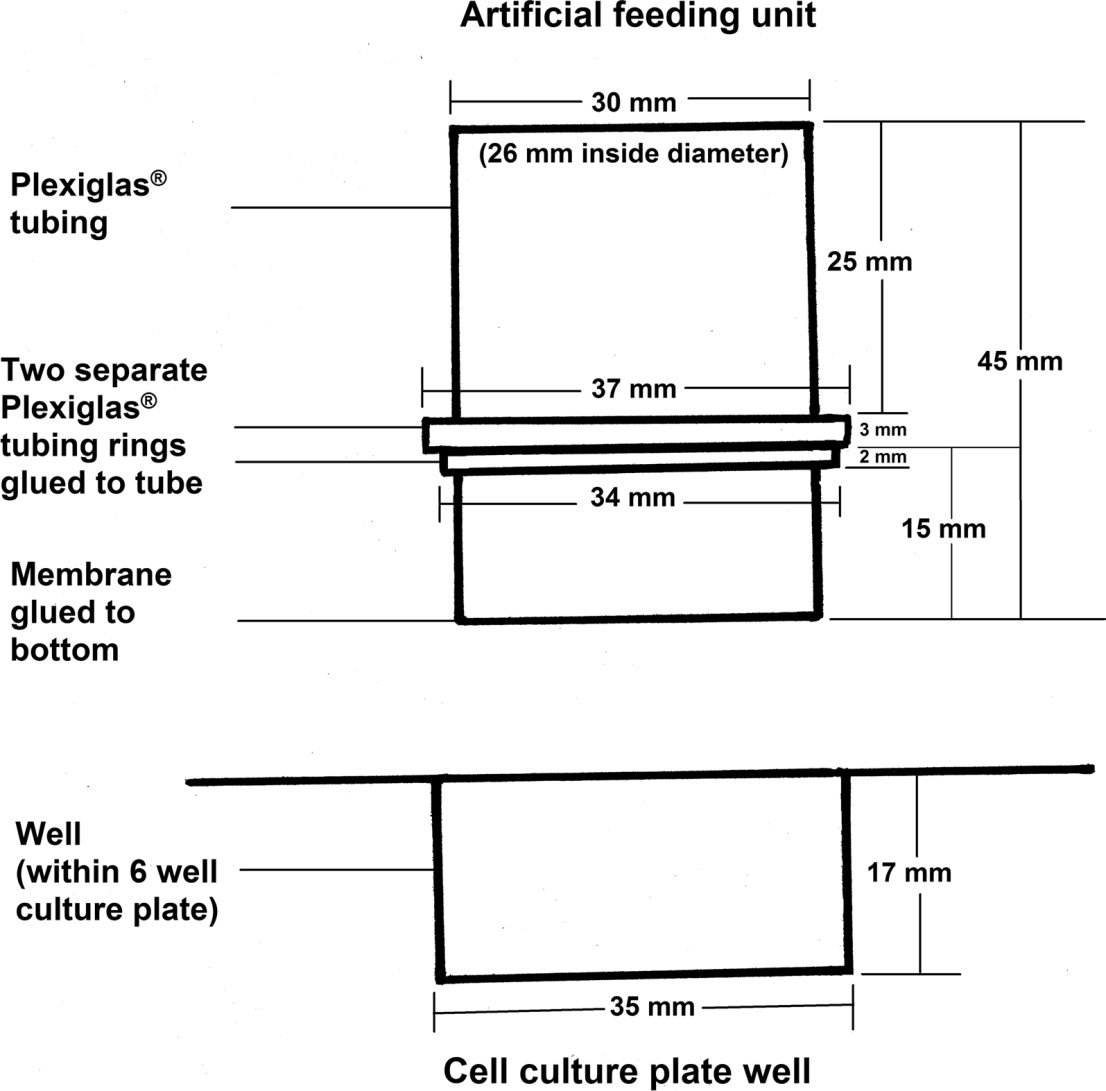
Schematic diagram of the feeding chambers used (as per Kröber & Guerin [23])

A total of 60 membrane feeding units in 6-well culture plates (35 mm diameter) containing bovine blood were used. The feeding chamber units, prepared according to Kröber & Guerin [23] were made of Plexiglas^®^ tubing (26 mm inside diameter, 2 mm wall thickness, 45 mm high; see Fig. 1).

**Fig. 2:**
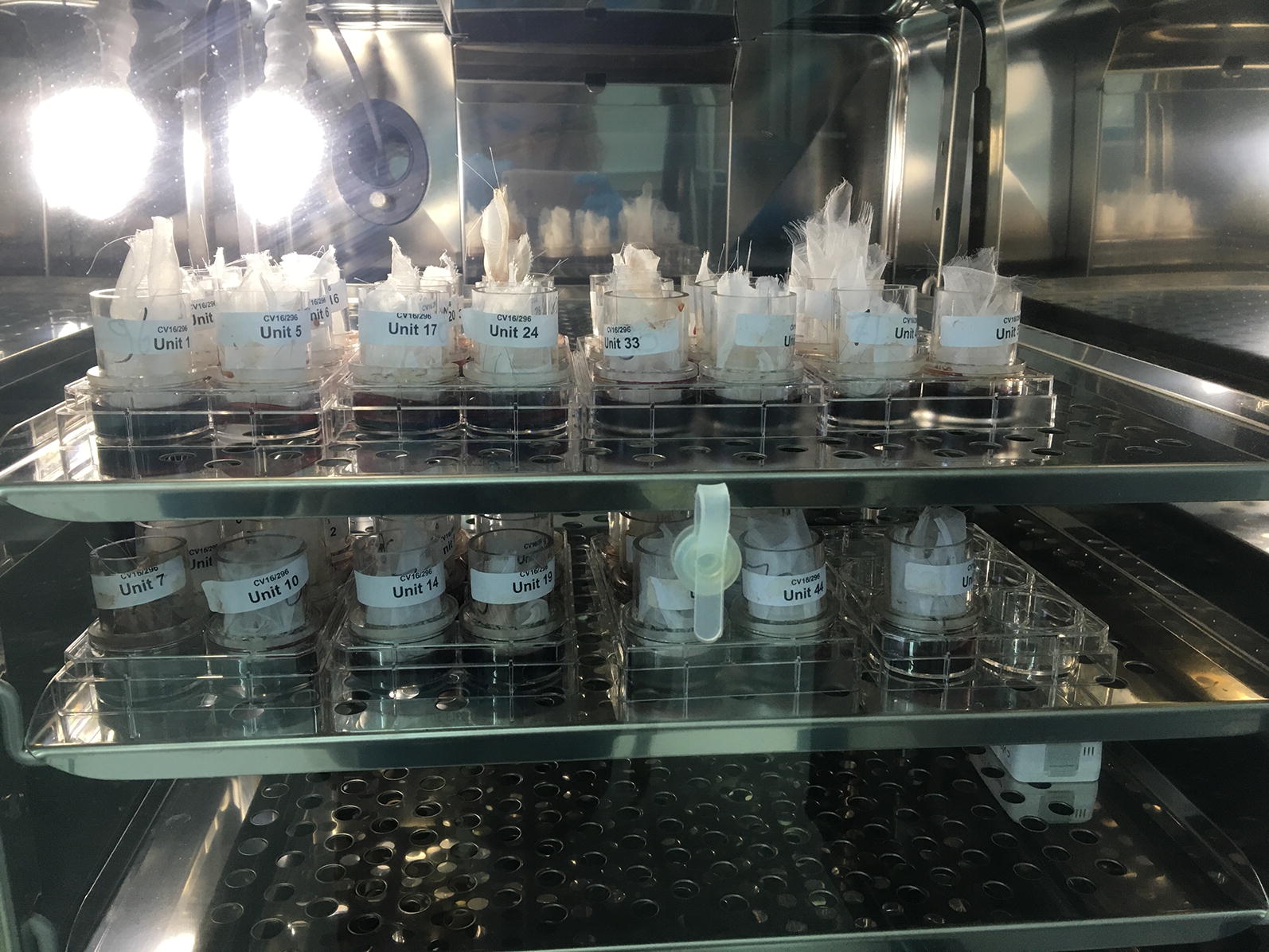
Example of feeding units in 6-well plates containing bovine blood in an incubator. Note the net covered stopper to preclude tick escape

These units were designed to fit into the wells of the 6-well culture plates so that the bottom of the feeding unit, which was covered by an artificial feeding membrane, was slightly elevated above the bottom of the plate. This allowed for the entire area of the feeding membrane to be covered with blood upon insertion into the well (Fig. 2). The artificial membrane was prepared as described in Fourie et al. [16].

**Fig. 3:**
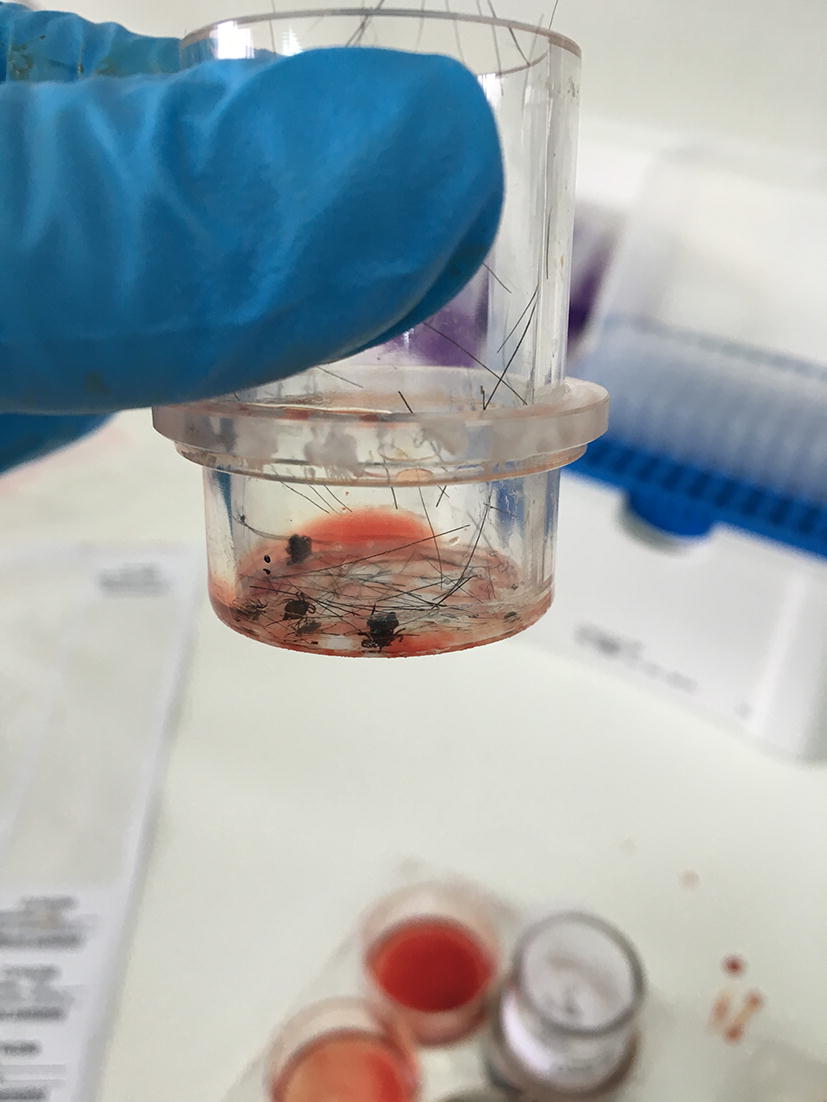
Ticks on the artificial membrane within the feeding unit after removal from the 6-well plate containing bovine blood (visible in the background). Also note the bovine hair clippings in the feeding unit

Cattle blood (from 3 donor cattle) was collected into Fenwal Blood Collection Bags [containing 2.45 g dextrose (monohydrate), 2.2 g sodium citrate (dihydrate) and 730 mg citric acid (anhydrous) per 100 ml of blood] on the day of tick seeding. Blood was stored at 4 °C until used for replacement of blood pools. Commercial gentamycin (5 µg/ml) and ATP (10 µm in the blood) were added to the blood just before it was filled into the wells. Approximately 3 ml of blood was required for each well. Prior to blood replacement, the required volume of blood, as well as saline used during the exchange process, was heated to approximately 37 °C. The chambers were kept in an incubator with a light/dark cycle of 18 h light:6 h dark. A thin layer of bovine hair, cut into pieces of approximately 4–7 mm was used to cover the membrane. A laboratory-bred strain of *I. ricinus* infected with *A. phagocytophilum* (predetermined *A. phagocytophilum* infectivity of 60%) was used to seed the chambers (see Fig. 3).

**Fig. 4:**
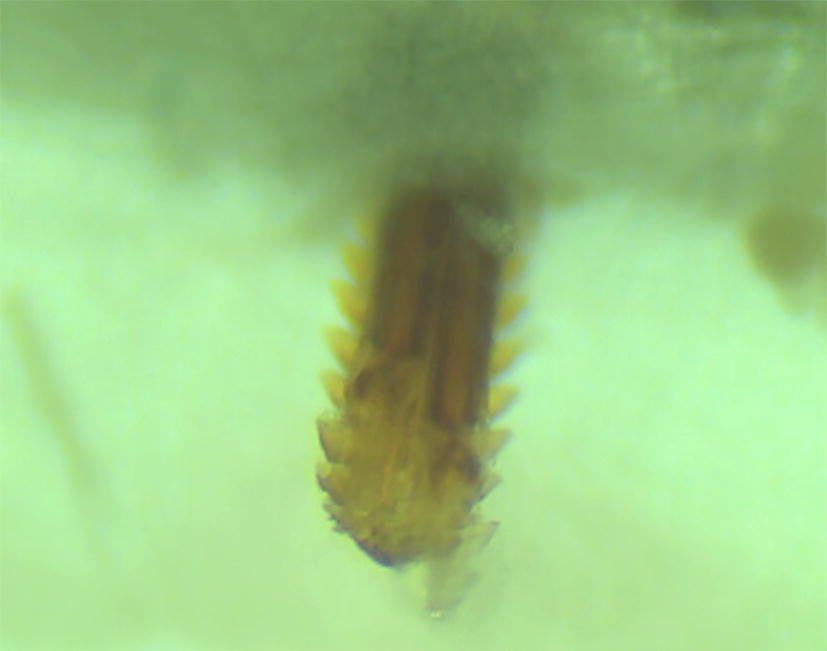
A tick hypostome as viewed from beneath the artificial membrane of the feeding unit after removal from a culture plate well containing cattle blood warmed to 37 °C

Feeding units were seeded with 10 ticks (5 males/5 females) and a stopper covered with the netting was placed above the ticks to prevent escape. Once ticks had been added to the feeding units, each unit was placed into a well containing blood (warmed to 37 °C), ensuring no air bubbles were present. Blood was replaced at least every 18 h or every 6 h once tick attachment was observed. This was done by adding fresh blood to a clean culture plate and by moving the feeding units to the clean plate. The membrane surface facing the blood was rinsed with warm sterile saline (37 °C) before placing the feeding unit into the fresh well. An image of the membrane, with a tick attached, is shown in Fig. 4.

Tick attachment observations were conducted on all chambers every 6 h. At the first time point where attachment was observed, all unattached female ticks were removed, the blood was sampled, the chamber placed into a fresh blood pool and the time recorded. The time at which the first tick attachment was observed, was considered the 6 h time-point.

At every subsequent assessment, the number of attached female ticks was recorded and detached female ticks were collected and stored in 70% ethanol. The blood pool was sampled (to allow analysis by qPCR for the presence of *A. phagocytophilum* DNA) and the tick chamber transferred to fresh blood pool. Assessments continued for a period of up to 72 h after first attachment, or until no further ticks were attached. After the final assessments, all ticks were removed and stored in 70% ethanol.

### qPCR analysis of the blood from the feeding chambers

Assessment of transmission of *A. phagocytophilum* to the blood in the feeding chambers required an alternative approach to ensure that each qPCR assessment contained significantly more of the target when compared to the traditional approach. For qPCR analysis on the blood collected from the artificial feeding chambers, frozen whole blood (up to 3 ml) was thawed and subjected to centrifugation for 10 min at 20,000 *rcf* at room temperature and the supernatant discarded. The pellet was resuspended in 1 ml 5% (w/v) ox bile ([24]; Sigma-Aldrich, St. Louis, USA) and incubated at room temperature for 10 min followed by centrifugation at 20,000 *rcf* for 10 min at room temperature. The supernatant was discarded, and the pellet again resuspended in 1 ml 5% (w/v) ox bile (Sigma-Aldrich) and incubated at room temperature for 10 min followed by centrifugation at 20,000 *rcf* for 10 min at room temperature. The supernatant was discarded and the pellet was resuspended in 200 µl PBS (Invitrogen, Carlsbad, USA) and subjected to genomic DNA isolation and qPCR detection as described above.

### Statistical analyses

Successful transmission of *A. phagocytophilum* by ticks to dogs was based on the detection of *A. phagocytophilum-*specific antibodies or DNA in dogs. Successful transmission of *A. phagocytophilum* by ticks feeding on artificial membranes was based on the detection of *A. phagocytophilum* DNA in the blood pools used for feeding. The first time point at which *A. phagocytophilum* was successfully detected was considered the minimum time required for the transmission of this bacterium by infected *I. ricinus* ticks *in vivo* and *in vitro*. No formal statistical analysis was conducted and the results are given descriptively.

## Results

### *Anaplasma phagocytophilum* strain

**Fig. 5:**
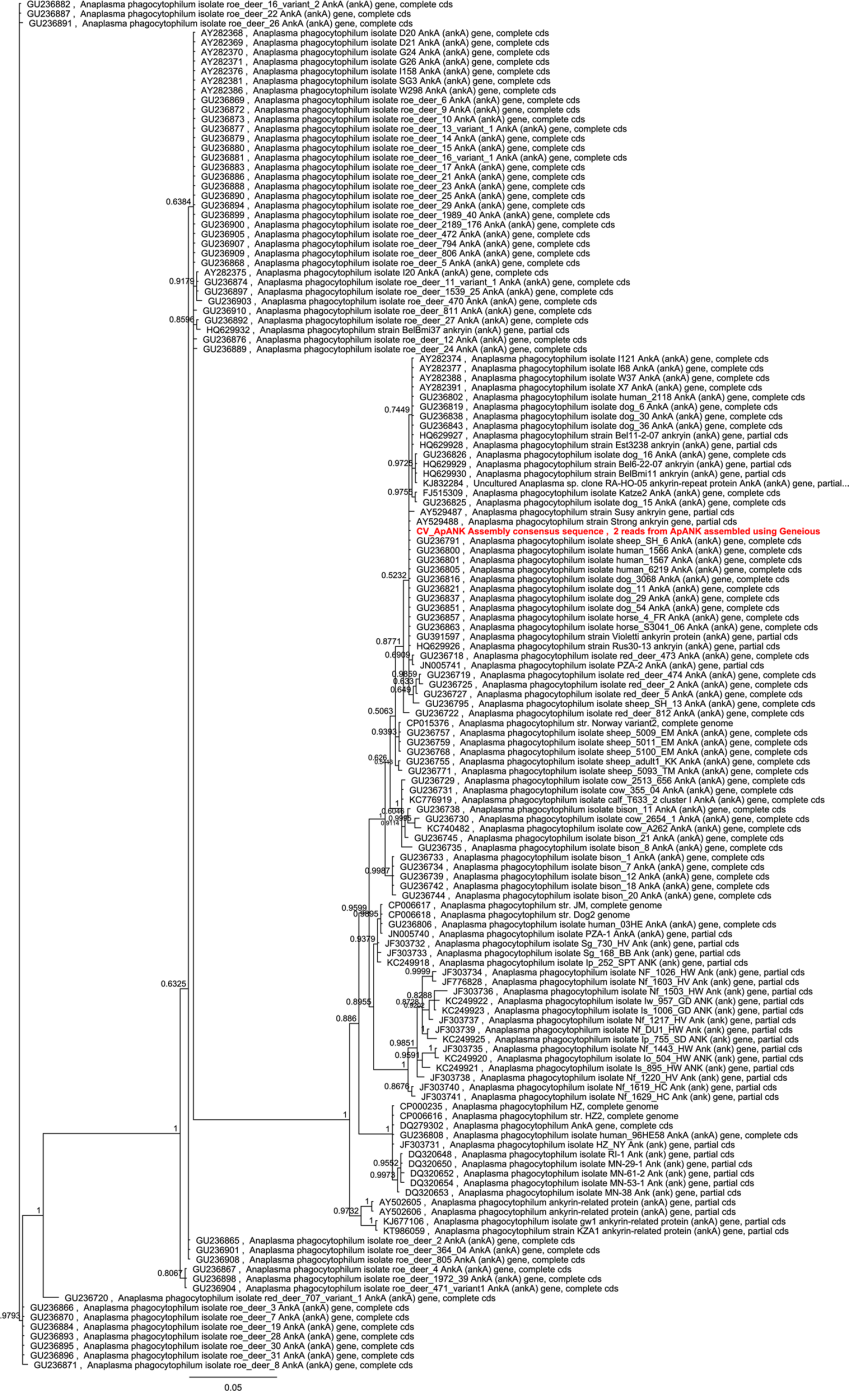
Phylogenetic tree based on amplification of the *ank* gene and sequencing of the PCR product

Amplification of the *ank* gene and sequencing of the PCR product revealed that this specific strain is closely related to other strains isolated from humans (USA and Slovenia), dogs, sheep and horses (Europe) based on the phylogenetic tree (Fig. 5). All these strains belong to the *ank* gene cluster I group [25].

### Dog study

All dogs included in the study were judged as clinically healthy by a veterinarian and were seronegative for *A. phagocytophilum*-specific antibodies. The mean number of attached female ticks in the infestation chambers (2 chambers per dog in Groups 1–5) ranged between 28.7–37.7 per dog. The arithmetic mean number of attached female ticks for Group 6 was 47.7 (see Table 1).

**Table 1:** Arithmetic mean number of female *Ixodes ricinus* ticks collected from the 6 study groups at the specific target times after infestation (Groups 1–5) or when fed until engorged (Group 6)

Hours post-infestation	Group	Mean no. of ticks
3	1	37.7
6	2	32.0
12	3	31.7
24	4	28.7
48	5	32.3
Females allowed to feed until engorged	6	47.7

Exposure of dogs to infected ticks was confirmed by conducting qPCR on pools of DNA extracted from attached female ticks removed from each dog. Each pool consisted of up to 5 ticks. The percentage of pooled DNA testing positive for *A. phagocytophilum* DNA ranged between 44.44–100%, confirming all dogs were exposed to infected ticks. No *A. phagocytophilum-*specific antibodies or DNA could be detected in any of the dogs in Groups 1–5. *Anaplasma phagocytophilum*-specific antibodies and DNA were detected in all 3 dogs in Group 6. In these dogs, *A. phagocytophilum* DNA was first detected in blood samples collected 7 days post-tick infestation in the first dog, 14 days post-infestation in the second dog and 21 days post-infestation in the third dog. All subsequent blood samples tested for these 3 dogs remained positive for *A. phagocytophilum* DNA. Seroconversion was first observed in 1 dog in Group 6, 28 days post-tick infestation and in the other two dogs 35 days post-infestation (see Table 2).

**Table 2:** Detection of *Anaplasma phagocytophilum* DNA and antibodies in blood samples taken from dogs in Groups 1–6 prior to tick infestation (Day 0) and weekly thereafter up to 63 days post-tick infestation

Group	Dog ID	Day (PCR/ SNAP® 4Dx® Plus)									
		0	7	14	21	28	35	42	49	56	63
1	216, 83, 184	−/−	−/−	−/−	−/−	−/−	−/−	−/−	−/−	−/−	−/−
2	121, 230, 513	−/−	−/−	−/−	−/−	−/−	−/−	−/−	−/−	−/−	−/−
3	863, 222, 075	−/−	−/−	−/−	−/−	−/−	−/−	−/−	−/−	−/−	−/−
4	833, 376, 851	−/−	−/−	−/−	−/−	−/−	−/−	−/−	−/−	−/−	−/−
5	895, 206, 226	−/−	−/−	−/−	−/−	−/−	−/−	−/−	−/−	−/−	−/−
6	495	−/−	−/−	−/−	+/−	+/−	+/+	+/+	+/+	+/+	+/+
	835	−/−	+/−	+/−	+/−	+/+	+/+	+/+	+/+	+/+	+/+
	859	−/−	−/−	+/−	+/−	+/−	+/+	+/+	+/+	+/+	+/+

*Key*: −, no DNA or antibodies detected; +, DNA or antibodies detected

In Group 6, where *A. phagocytophilum* infected female adult ticks were left on the dogs until engorged, *A. phagocytophilum* MSP2 copy numbers detected by qPCR increased over time (Days 0 to 28 for 1 dog and Days 0 to 28 for the remaining 2 dogs; see Table 3).

**Table 3:** Relative copy number of the MSP2 target in blood collected from dogs in Group 6 (ticks fed on dogs until engorged)

Group 6 Dog ID	Relative copy number of the MSP2 target on:						
	Day 0	Day 7	Day 14	Day 21	Day 28	Day 35	Day 42
495	0	8	0	304	719	274	130
835	0	15	5588	5135	1263	431	2108
859	0	0	804	3222	234	74	115

The body temperatures for all dogs ranged between 36.5–39.4 °C, which was within the range considered normal for dogs. No clinical symptoms associated with acute canine granulocytic anaplasmosis were observed in any of the dogs.

### *In vitro* study

**Fig. 6:**
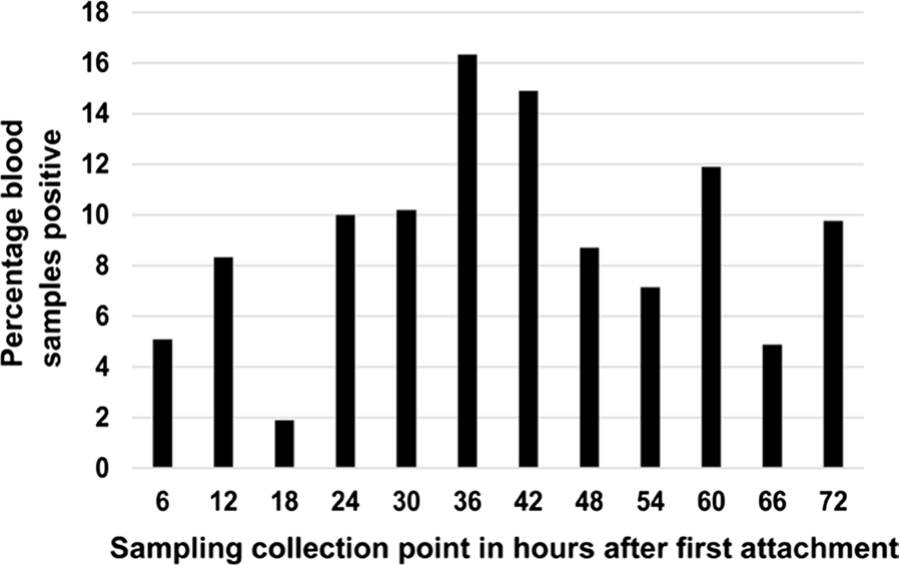
Percentage of blood samples in which *Anaplasma phagocytophilum* DNA was detected (out of total number tested) at each of the respective time points during the *in vitro* experiment

Tick attachment in the 60 chambers over the 72 h ranged between 20–60% (i.e. 1–3 female ticks attached in each chamber) with all 60 chambers having at least 1 attached female tick. The speed at which at least one tick attached in each chamber ranged from 6–18 h post-seeding with attachment observed in 56.7% of chambers at 6 h. By 72 h after observing the first attachment, 66.7% of the chambers still contained at least 1 attached tick. *Anaplasma phagocytophilum* DNA was detected in the blood collected from 3 (5%) out of 60 chambers at 6 h (defined as the time point where the first attached tick was observed) with the highest number of positive samples (8 out of 49; 16.3%) in chambers with ticks still attached at 36 h (Fig. 6).

Detection of *A. phagocytophilum* in blood samples collected and replaced every 6 h from individual chambers were intermittent, with no consistent detection of DNA being observed from feeding units with ticks attached for longer than 18 h after first attachment.

## Discussion

The experimental infection of sheep with an *A. phagocytophilum* strain (“TIBA strain”) isolated from a clinical case (dog) enabled the successful infection of multiple tick batches by feeding *I. ricinus* nymphs until repletion on a bacteremic host. The experimental infection of ticks yielded an infectivity between 21–60% in different batches and was sufficient to demonstrate transmission of *A. phagocytophilum* bacterium *in vivo* in dogs and *in vitro* using an artificial feeding system. Furthermore, the temporal increase in copy number of the *A. phagocytophilum* MSP2 target in DNA isolated from whole blood obtained from dogs in Group 6 demonstrates that *A. phagocytophilum* was able to multiply in blood over time and that the bacteria were thus alive. This further validates the success of the model employed. Transmission of *A. phagocytophilum* bacterium was only detected based on qPCR and specific antibody assay (SNAP® 4Dx® Plus test) in dogs that infected ticks had fed on until engorged. No infection was detected in dogs when ticks were removed from 3 up to 48 h post-infestation. In contrast, *A. phagocytophilum* DNA was observed as early as 6 h post-feeding in the blood pools that infected ticks had fed on *in vitro*. Using an ox bile pretreatment of the blood from the feeding chambers, resulted in the reduction host DNA contamination of the isolated DNA yielding a 15-fold increase in effective blood volume that could be assessed during qPCR and an increased detection sensitivity compared to the untreated methodology recommended by the nucleic acid isolation kit manufacturer. Taking this, as well as the > 100 copies of the MSP2 qPCR targets present per *A. phagocytophilum* genome into account, this extremely sensitive approach enabled detection of *A. phagocytophilum* DNA present in the blood pools [26]. The seemingly contradictory results based on qPCR observed *in vivo *in dogs and *in vitro* using artificial feeding units could potentially be attributed to the increased sensitivity of the assay used to assess the blood pools from the *in vitro* test. Moreover, transmission of *A. phagocytophilum* by ticks has been demonstrated *in vitro* to take place soon after attachment. As a result, establishment and detection of an infection in dogs might be dependent on a minimum *A. phagocytophilum* bacteria dose inoculated to achieve infection and consequent detection on the multiplication of *A. phagocytophilum* bacteria in the host, until the detection threshold for qPCR is reached. In the present study, no seroconversion was observed in dogs challenged with infected ticks when removed within 48 h after infestation, although transmission of *A. phagocytophilum* bacteria should have occurred. Hodzic et al. [18] demonstrated that, although they could not accurately determine the tick-borne infectious dose, infection with *A. phagocytophilum* is dose-dependent and that relatively high doses of organisms appear to be needed to infect a mouse. This is also the case for other related organisms such as *Ehrlichia risticii*, *E. canis*, *Rickettsia australis* and *Rickettsia conorii* where dosage studies have demonstrated that the innate defense mechanisms of hosts can protect against or eliminate low dose inoculation and it is only at higher doses that infection and disease is established [27, 28]. Moreover, it has also been shown that replication of *A. phagocytophilum* bacteria occurred within feeding ticks, also increasing the effectiveness of transmission and ultimately the speed at which the minimum inoculation dose needed for infection in the host is reached [18]. Considering the results of the present study, as well as the dosage studies done on other hosts like mice, infection with *A. phagocytophilum* in dogs seems also to be dose-dependent and relatively high doses of organisms appear to be needed for the establishment of infection. More research is, however, needed to determine the minimum infective dose for *A. phagocytophilum* in dogs.

## Conclusions

Transmission of *A. phagocytophilum* by *I. ricinus* ticks starts within a few hours after attachment, but establishment of infections in dogs is apparently dependent on a minimum inoculation dose that was only observed in the present study when ticks attached for greater than 48 h. These findings highlight the need for acaricidal products to exert a repellent and/or rapid killing effect on ticks to forestall or interrupt transmission of *A. phagocytophilum* and ultimately prevent clinical infection and disease in dogs.

## Abbreviations

DMSO: dimethyl sulfoxide
DNA: deoxyribonucleic acid
EDTA: ethylenediamine tetraacetic acid
ID: identification
PCR: polymerase chain reaction
